# Supplementary material for: Complexome profiling of the Chlamydomonas psb28 mutant reveals TEF5 as an early PSII assembly factor
Source: Plant Cell. 2025 Mar 17;37(6):koaf055. doi: 10.1093/plcell/koaf055 (PMC12164590; doi:10.1093/plcell/koaf055)
Supplement: koaf055_Supplementary_Data [file koaf055_supplementary_data.zip › psb28_interactive.zip!/plots/intensity/qualityPlots/peptides/PCR1_Cre03.g154550.t1.1.html]

### Cre03.g154550.t1.1

Trivial name: PCR1  
SeqCoverage [median]: 34.90  
Mass: 35.95 kDa  
  
 

| Sequence | MissedCleavage | Mass [Da] | wtIntensity | muIntensity | Score |
| --- | --- | --- | --- | --- | --- |
| AAFMNAVVAATDR | 0 | 1335.66 | 872.90 | 2114.50 | 112.13 |
| ANADDETLVR | 0 | 1102.53 | 0.00 | 1480.79 | 76.83 |
| DIAQALAAQTVLGSAK | 0 | 1555.86 | 2715.00 | 0.00 | 131.53 |
| IGFLGSGQMAEALAR | 0 | 1519.78 | 3021.53 | 6816.18 | 102.87 |
| IYTVDEK | 0 | 866.44 | 0.00 | 536.34 | 97.07 |
| LVEAAGPDAHVVR | 0 | 1332.71 | 0.00 | 463.81 | 61.53 |
| MVMETGTHPGALKDMVTSPAGTTIAGVHELER | 1 | 3335.63 | 0.00 | 2621.09 | 56.71 |
| SFGATPYESNADVAR | 0 | 1583.72 | 2505.26 | 2525.89 | 129.34 |
|  |  |  | 9114.7 | 16558.6 |  |
